# [Retracted] Overexpression of microRNA‑155 alleviates palmitate‑induced vascular endothelial cell injury in human umbilical vein endothelial cells by negatively regulating the Wnt signaling pathway

**DOI:** 10.3892/mmr.2025.13559

**Published:** 2025-05-06

**Authors:** Yanping Zhao, Wenquan Rao, Yanna Wan, Xinglong Yang, Guorong Wang, Jun Deng, Min Dai, Qiang Liu

Mol Med Rep 20: 3527–3534, 2019; DOI: 10.3892/mmr.2019.10623

Following the publication of this paper, it was drawn to the Editors’ attention by a concerned reader that the majority of the flow cytometric assay data shown in Fig. 4A on p. 3531 were strikingly similar to data appearing in different form in other articles written by different authors at different research institutes that had already been published elsewhere prior to the submission of this paper to *Molecular Medicine Reports* (several of which have already been retracted).

In view of the fact that the abovementioned data had already apparently been published previously, the Editor of *Molecular Medicine Reports* has decided that this paper should be retracted from the Journal. The authors were asked for an explanation to account for these concerns, but the Editorial Office did not receive a reply. The Editor apologizes to the readership for any inconvenience caused.

